# Simultaneous and interleaved acquisition of NMR signals from different nuclei with a clinical MRI scanner

**DOI:** 10.1002/mrm.26056

**Published:** 2015-11-26

**Authors:** Martin Meyerspeer, Arthur W. Magill, Andre Kuehne, Rolf Gruetter, Ewald Moser, Albrecht Ingo Schmid

**Affiliations:** ^1^ Center for Medical Physics and Biomedical EngineeringMedical University of ViennaAustria; ^2^ MR Centre of Excellence, Medical University of ViennaAustria; ^3^ Laboratory for Functional and Metabolic Imaging, École Polytechnique Fédérale de LausanneLausanneSwitzerland; ^4^ Department of RadiologyUniversity of LausanneLausanneSwitzerland; ^5^ Department of RadiologyUniversity of GenevaGenevaSwitzerland; ^6^ Present address: Institute of Neuroscience and Medicine 4Forschungszentrum Jülich GmbHJülichGermany

**Keywords:** X‐nucleus, interleaved, simultaneous, multinuclear, MRS, MRI

## Abstract

**Purpose:**

Modification of a clinical MRI scanner to enable simultaneous or rapid interleaved acquisition of signals from two different nuclei.

**Methods:**

A device was developed to modify the local oscillator signal fed to the receive channel(s) of an MRI console. This enables external modification of the frequency at which the receiver is sensitive and rapid switching between different frequencies. Use of the device was demonstrated with interleaved and simultaneous ^31^P and ^1^H spectroscopic acquisitions, and with interleaved ^31^P and ^1^H imaging.

**Results:**

Signal amplitudes and signal‐to‐noise ratios were found to be unchanged for the modified system, compared with data acquired with the MRI system in the standard configuration.

**Conclusion:**

Interleaved and simultaneous ^1^H and ^31^P signal acquisition was successfully demonstrated with a clinical MRI scanner, with only minor modification of the RF architecture. While demonstrated with ^31^P, the modification is applicable to any detectable nucleus without further modification, enabling a wide range of simultaneous and interleaved experiments to be performed within a clinical setting. Magn Reson Med 76:1636–1641, 2016. © 2015 The Authors. Magnetic Resonance in Medicine published by Wiley Periodicals, Inc. on behalf of International Society for Magnetic Resonance in Medicine. This is an open access article under the terms of the Creative Commons Attribution License, which permits use, distribution and reproduction in any medium, provided the original work is properly cited.

## INTRODUCTION

Magnetic resonance imaging and spectroscopy are powerful tools for investigating human anatomy and physiology in vivo. While the majority of studies and clinical routine MRI focus on ^1^H, other nuclei are also detectable, and all major hardware manufacturers provide system options to enable detection of these nuclei using clinical scanners. Nonproton, or X‐nuclear, studies give access to information not available from proton detection alone. For example, ^31^P is used to investigate metabolism via high energy phosphates 1, 2, ^13^C provides information about glucose and glycogen metabolism 3, 4, 5, and ^23^Na may be used to assess the viability of healthy and tumorous tissue 6, 7, 8 or cartilage defects 9.

Most in vivo multinuclear MR experiments essentially involve a series of mono‐nuclear acquisitions. However, simultaneous detection of signals from more than one nuclear species is possible 10, 11, 12, offering a reduction in total measurement time 13, 14. In practice, simultaneous acquisition is difficult because field gradients always act on both nuclei so that, for example, images acquired simultaneously from two different nuclei will have different fields of view, scaled by the respective gyromagnetic ratios 15. An alternative approach is to rapidly interleave acquisitions of two nuclear species 16, 17, 18, which offers similar benefit to simultaneous acquisition but without this intrinsic challenge. Interleaving also allows one nucleus to be sampled more frequently than the other 18, 19, 20, which is important for optimal signal‐to‐noise ratio (SNR) per unit time, given the potentially very large differences in intrinsic sensitivity and *T*_1_ between ^1^H and X.

Simultaneous or interleaved acquisition can be exploited to acquire complementary data from transient states that are difficult or even impossible to repeat precisely, e.g., in patients, or when repetition of the experiment affects physiological parameters. For example, interleaved multinuclear ^31^P MRS and ^1^H MRI have been used to investigate the regulation of oxidative and glycolytic metabolism in skeletal muscle during a single exercise 18, 20, 21. Recently developed methods for quantitation of phosphorylated metabolites 22, 23 and pH 24 by ^31^P MRI and ^31^P MRS 25 would benefit by combination with interleaved measurements of perfusion and blood oxygenation by ^1^H MRI 26. Further, coregistration problems between ^1^H and X‐nuclear data sets could be alleviated and promising applications, such as dynamic shimming or ^1^H‐based motion correction of X‐nuclear data 12, require interleaved acquisition of ^1^H imaging data. However, most of these prior works on multinuclear simultaneous and interleaved detection have been accomplished on research systems or with significant modification to the spectrometer.

To run simultaneous or interleaved dual‐nuclear experiments, the MRI system must be capable of exciting the sample, and recording the resulting NMR signals, at the two different frequencies. Transmit pulses must be generated simultaneously or in quick succession. Most multinuclear‐capable MRI systems already have this capability, intended to be used for nuclear Overhauser enhancement (nOe), polarization transfer, and J‐decoupling. Also, the RF probe must be simultaneously tuned to both frequencies of interest. A standard dual‐resonant probe 27, 28, 29 may be used here, including those commercially available. Finally, the MRI console must be able to process and digitize signals at two different frequencies or, for interleaved acquisition, rapidly switch between two different acquisition frequencies. It is this last part that is generally not possible using a standard clinical MRI scanner, and that we address in this work.

## METHODS

### Functional Principle

MR signals can occur over a wide range of frequencies, typically on the order of tens to hundreds of megahertz. Rather than digitizing NMR signals directly at the Larmor frequency, most MRI spectrometers use the superheterodyne principle to first convert the signal down to a lower frequency of the order of a few megahertz 30. This is done by mixing the received signal with a reference signal, called the local oscillator (LO), to produce an output at the frequency difference between the original signal and the LO, known as the intermediate frequency (IF). By adjusting the LO, which is generated by the system synthesizer, the IF can always be set to a fixed frequency, independent of the frequency of the NMR signal. The signal is then directly digitized at the IF by the analogue‐to‐digital converter (ADC).

**Figure 1:**
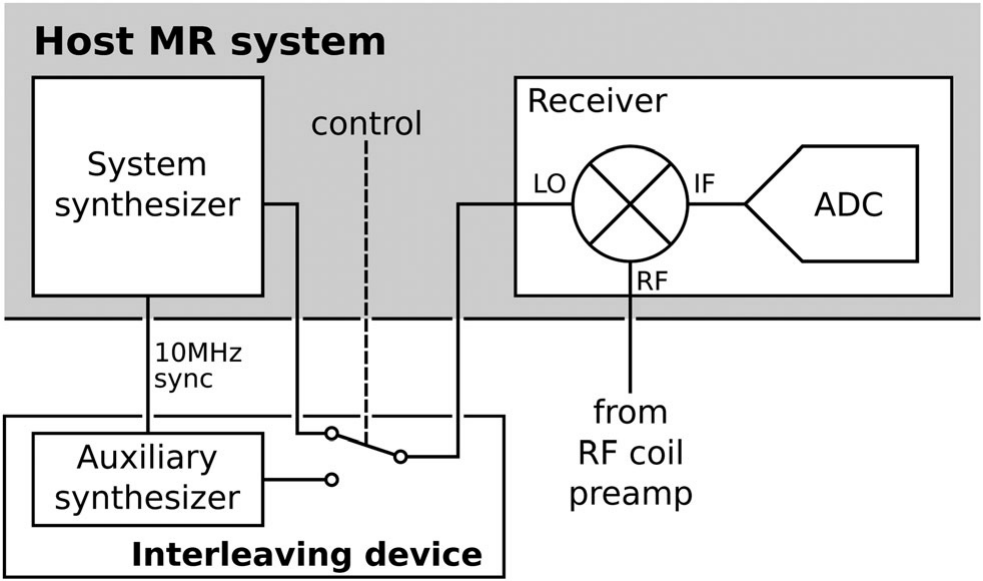
Schematic diagram showing the RF pathway modification. The interleaving device is inserted between the system synthesizer and LO input of the receiver. In passive mode, the system LO is passed through the interleaving device, while in active mode, a replacement LO signal is generated by the auxiliary synthesizer, which is fed to the receiver in place of the system LO. The interleaving device is switched between passive and active mode via a control signal generated by the scanner and controlled via the pulse sequence.

By externally modifying the LO, the frequency at which the spectrometer is sensitive can be manipulated. This is the principle behind the operation of the interleaving device, built to our specification by Pure Devices (Würzburg, Germany), shown schematically in Figure 1. It is inserted into the system receive chain between the synthesizer and the receivers. By default, the interleaving device rests in the passive state, passing the system LO from input to output. All system adjustments and mononuclear experiments can be performed in the normal manner when the interleaving device is in this state. The device is activated by a digital control signal provided by the MRI console and controlled via the pulse sequence. Once active, the interleaving device replaces the system LO with a signal generated by an auxiliary synthesizer. This synthesizer has a frequency resolution of 1 kHz and is synchronized to the MRI scanner via a console‐provided 10 MHz reference signal to ensure frequency and phase stability relative to the console. The frequency of the auxiliary synthesizer is set using an external computer via a Universal Serial Bus (USB) connection. When the interleaving device is active, the frequency of the signal digitized by the scanner is determined by the auxiliary synthesizer, rather than the console.

The frequency synthesizer of the MRI console used in this work has a resolution of 2.5 MHz, limiting how accurately the LO can be offset from the Larmor frequency, resulting in different IFs for different nuclei. The ADC and RF stage following the mixer have a much wider bandwidth than the MR signal. Provided that the IF offset plus the bandwidth of the MR signal fits inside this wider bandwidth, the signal may be recorded at full fidelity. The frequency offset is then removed in digital postprocessing. The offset frequency used in this digital stage is not user‐configurable, so the frequency of the auxiliary synthesizer must be set to reproduce the IF of the nuclei that the console is configured to detect. For example, to digitize a ^31^P signal at 7 T (*f*_0_ = 120.32 MHz), the console provides a LO at 122.50 MHz to give an IF of 2.18 MHz. To record a ^1^H signal (*f*_0_ = 297.22 MHz), the interleaving device LO must be set to 299.41 MHz to reproduce the same IF. The receive bandwidth of the final signal is controlled per ADC event via the dwell time as a pulse sequence parameter and can be selected independently for each nucleus.

The receive channels of the MRI system used in this work are grouped into four banks of eight channels, each bank having a separate LO input. For interleaved acquisition, the frequency of any or all banks of receive channels can be switched. For simultaneous acquisition at two frequencies, at least one bank must continue to operate unchanged.

The interleaving device only modifies the receive chain of the scanner. The RF coil and its interface to the scanner require no customization. Any coil capable of X and ^1^H acquisition is suitable for interleaved acquisition, and any coil capable of heteronuclear decoupling can be used for simultaneous acquisition. The MRI console is already capable of generating transmit pulses at the Larmor frequency and at a second “decoupler” frequency, within a single pulse sequence. This feature is intended for nOe and J‐decoupling, but can be used to generate excitation pulses for a second nuclear species.

The safety‐critical transmit chain is not modified in any way, so the scanner RF safety system, including SAR monitoring, continues to operate in the normal manner. The interleaving device, therefore, has no impact on system safety or safety monitoring.

### MR Experiments

To evaluate the function and performance of the interleaving device, spectroscopy and imaging experiments were conducted on a Siemens Magnetom 7 T (Siemens Medical, Erlangen, Germany). All experiments on the 7 T system were performed using an in‐house built RF probe, consisting of two ^1^H and three ^31^P transceive elements, shaped to a half cylinder (*d* = 14 cm, *l* = 10 cm) 29. The test object was a spherical phantom containing 500 mL aqueous phosphate solution (K_2_HPO_4_, 100 mmol/L). Functionality was also tested on a Siemens Tim Trio, a 3 T MRI system equipped with a concentric ^31^P/^1^H single loop coil (*d* = 10 cm, Rapid Biomedical, Würzburg, Germany).

**Figure 2:**
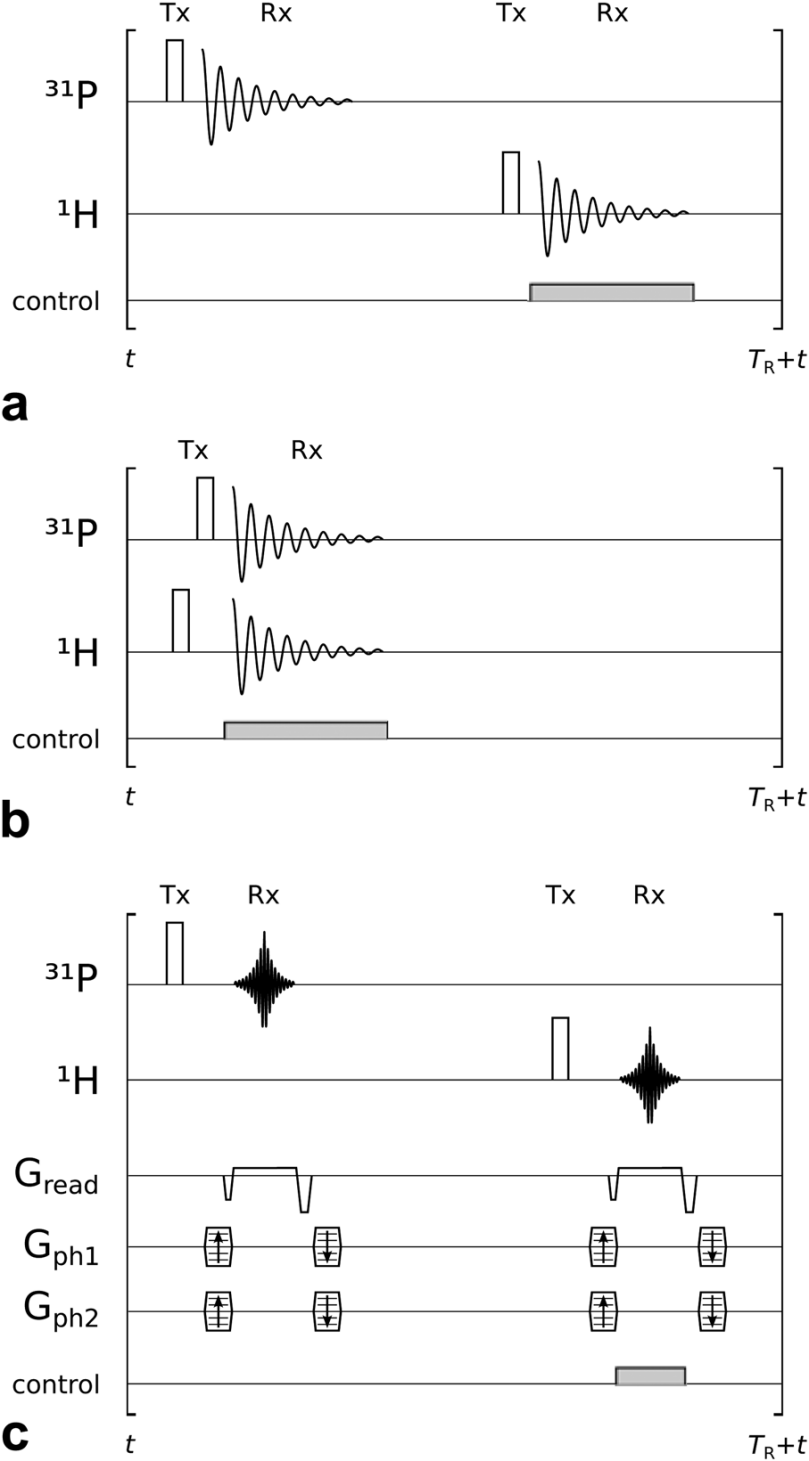
Pulse‐acquire sequences, for (**a**) interleaved and (**b**) simultaneous acquisition of ^1^H and ^31^P spectra; (**c**) shows a 3D gradient‐echo imaging sequence, which alternately acquires k‐space lines for ^1^H and ^31^P.

To examine the interleaving device's influence on SNR and signal amplitude in NMR experiments as directly as possible, several series of pulse‐acquire ^1^H and ^31^P spectra were compared. ^1^H‐only and ^31^P‐only measurements were conducted without the interleaving device present, and with the interleaving device connected but passive (i.e., passing the system LO to the receivers). Interleaved and simultaneous multinuclear acquisitions were then made using the interleaving device with the sequences shown in Figure 2a and b. The interleaving device is only active during ^1^H reception. For simultaneous multinuclear acquisition, the LO fed to the receivers recording the ^1^H signal is modified by the interleaving device, while the receivers recording the ^31^P signal use the system LO unmodified. As the MRI console is not able to generate simultaneous RF pulses at two different frequencies, fast sequential excitation of both nuclei was used 31, as shown in Figure 2b. To eliminate the influence of nOe 18, experiments were repeated with the ^1^H transmit pulse amplitude set to zero for ^31^P data quantification.

Because both the system and the auxiliary LO are running phase continuously, a constant phase will be accumulated in every consecutive acquisition of data (i.e., each k‐space line or averaging step, spaced by *T*_R_) that are subject to mixing with the interleaving device's LO. This phase accrual can be accounted for by prospectively incrementing the phase of the ^1^H acquisition by

(1) $$\Delta \varphi =(f_{{}^{1}}{}_{H}-f_{X})\cdot T_{R},$$

where $f_{{}^{1}}{}_{H}$ and *f*_X_ are the respective Larmor frequencies and *T*_R_ is the repetition time.

For reproducible SNR quantification, 50 spectra were acquired in each state (2048 complex points, 5000 Hz spectral bandwidth), using a repetition time of 2 s. SNR was quantified in the frequency domain as peak amplitude divided by the standard deviation of data points from an artefact‐free flat baseline region of the spectra. For ^1^H free induction decays, small nutations were used (∼0.1° near the coil), resulting in a constant signal amplitude throughout each measurement series and a flat spectral baseline. Phosphorous free induction decays, with inherently lower SNR, were excited with higher flip angles (∼70°). The first 10 spectra were excluded from each dataset to ensure that the system had reached steady‐state.

Images were also acquired to demonstrate use of the interleaving device with a more complicated acquisition sequence involving faster frequency switching and the use of field gradients. A 3D gradient‐echo sequence was modified to alternately acquire k‐space lines for each nucleus (Fig. 2c). The echo time and effective repetition time for each nucleus was *T*_E_ = 3.8 ms and *T*_R_ = 16 ms. Equal gradient amplitudes were used for both nuclei, resulting in 2.47 times ( $\gamma_{{}^{1}}{}_{H}/\gamma_{{}^{31}}{}_{P}$) larger voxels and field of view for the ^31^P images than for ^1^H images, helping to compensate for the lower sensitivity and abundance of ^31^P. The nominal voxel size was 3.1×3.1×12.4 mm^3^ for the ^31^P images and 1.25×1.25×5 mm^3^ for the ^1^H images, with the same matrix size of 128×128×24 for both nuclei. Four averages were acquired in interleaved and ^31^P‐only scans, leading to a total measurement time of 3 min 17 s. For ^1^H MRI, unaveraged data are shown. Images were reconstructed offline using software written in‐house in the Perl Data Language (PDL, http://pdl.perl.org).

## RESULTS

NMR data were successfully collected with interleaved and simultaneous multinuclear acquisitions on a Magnetom 7 T and a Tim Trio 3 T system. No adverse effects of the interleaving device on signal amplitude, SNR or phase stability were found. The switching time of the interleaving device, measured using an oscilloscope (TDS 3052, Tektronix, Beaverton, OR), was found to be within 10 μs of the control signal changing state.

**Figure 3:**
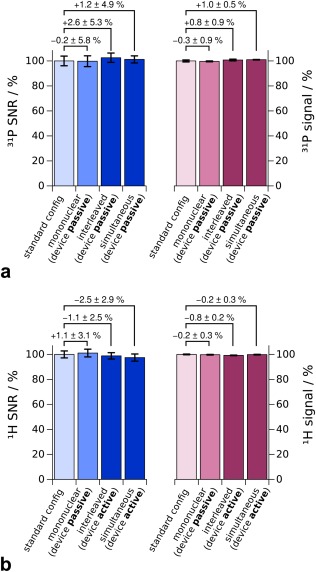
Signal‐to‐noise ratio and signal amplitude of (a) ^31^P and (b) ^1^H pulse‐acquire experiments using a test object. Performance with the scanner in the standard configuration was not degraded when the interleaving device was added in passive mode, and when it was used to enable interleaved or simultaneous acquisitions. Note that the interleaving device is only ever active during ^1^H acquisitions. For interleaved acquisitions, the device is always passive during ^31^P readout, while for simultaneous acquisitions ^31^P is measured on a receive bank not connected to the device.

The results of the performance tests using pulse‐acquire experiments at 7 T are shown in Figure 3. Measurement precision was better than ±1 % for the signal amplitude for both nuclei, approximately ±6 % for ^31^P SNR, and ±3 % for ^1^H SNR. Looping the system LO through the interleaving device in the passive state made no measurable difference to SNR or signal intensity in free induction decays obtained for either nucleus. For simultaneous and interleaved ^31^P/^1^H acquisitions, an nOe‐induced increase of ^31^P signal and SNR on the order of 10% was observed when ^1^H magnetization was excited 18. SNRs obtained for ^31^P during interleaved and simultaneous measurements without ^1^H excitation were identical to those measured in mononuclear experiments without the interleaving device connected, to within measurement precision, while the differences in signal amplitudes were below 1%. Exciting ^31^P magnetization produced no measurable nOe in ^1^H spectra. Spectra used to quantify ^1^H signal intensity and SNR in simultaneous and interleaved acquisitions were measured in the presence of ^31^P excitation and showed no change in signal intensity or SNR when compared with mononuclear acquisitions made without the interleaving device present. In summary, the interleaving device did not affect the SNR or amplitude of ^31^P or ^1^H signals in any of the above measurements.

**Figure 4:**
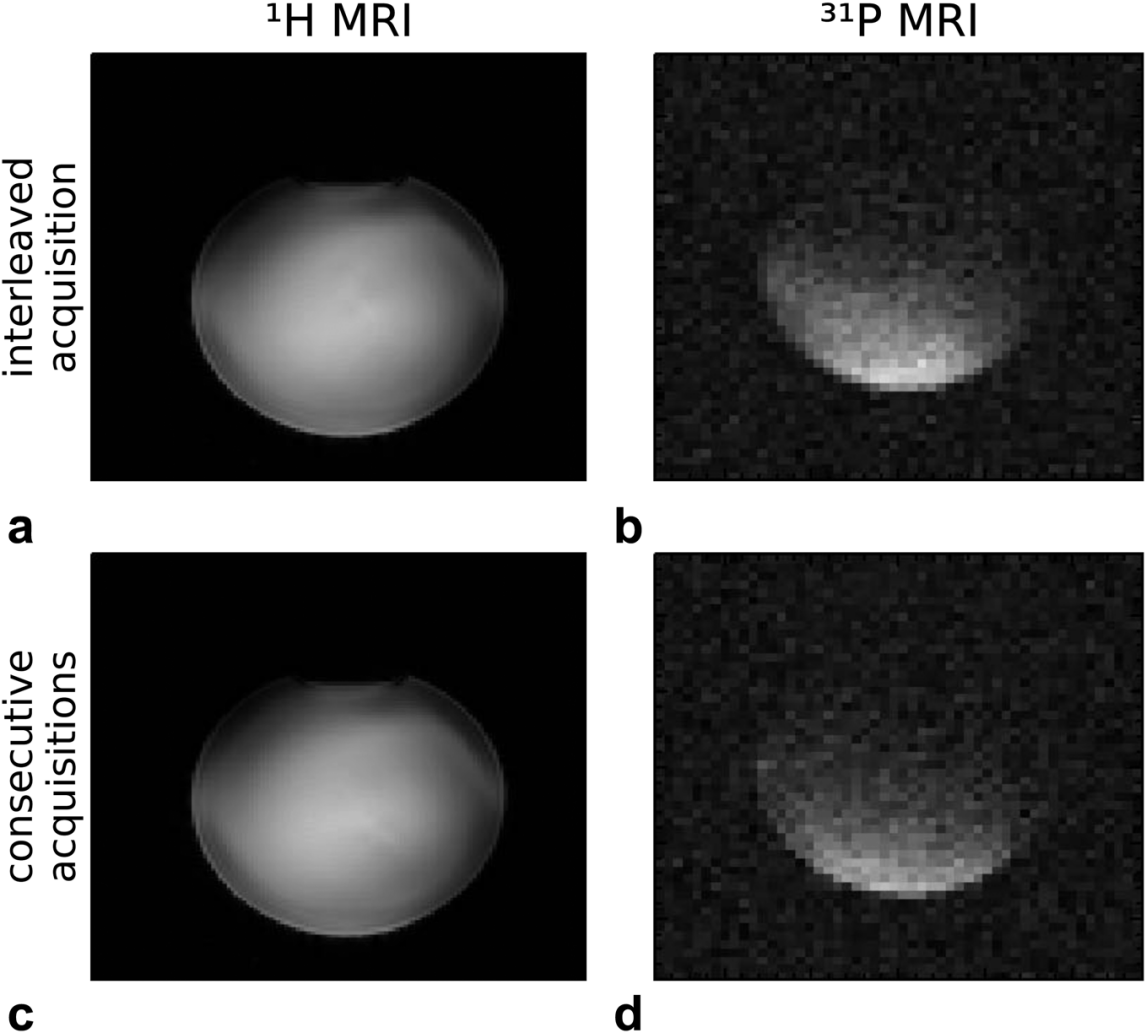
^1^H (**a**, **c**) and ^31^P (**b**, **d**) images acquired with (a, b) and without (c, d) interleaving. Images in the top row were acquired in a single experiment. Images in the bottom row were acquired in two separate experiments, using the same sequence, but with transmission at the unused frequency omitted, to demonstrate the effect of nOe. ^31^P images were cropped to have the same FOV as ^1^H images. The increased brightness of (b) relative to (d) can be ascribed to nOe from the ^1^H excitation pulses.

Images acquired at 7 T are shown in Figure 4. No difference is seen between ^1^H images acquired with and without interleaving. An increase in signal intensity is seen in the ^31^P image with interleaving, relative to the image without, due to nOe from the ^1^H pulses. It was verified that the larger ^31^P FOV in interleaved acquisitions did not have an effect on SNR, ^31^P images were also acquired with the same voxel size but with a reduced matrix size of 52×52×10, corresponding to the same FOV as ^1^H images. The number of averages was increased to 24, for comparable total measurement time (3 min 20 s).

A constant phase accrual was found between consecutive *T*_R_ steps in ^1^H data. The phase increment between 30 free induction decays acquired in a single scan was linearly *T*_R_‐dependent ( $R^{2}\geq 0.9999$) and could be corrected by introducing an incremental offset to the receiver phase for ^1^H acquisitions using Eq. (1). ^31^P data, acquired using the system LO routed via the interleaving device, showed no phase drift.

## DISCUSSION AND CONCLUSION

In this work, we have demonstrated a simple hardware modification to a clinical MRI scanner that enables interleaved and simultaneous acquisition of signals from two different nuclei. Using ^31^P and ^1^H, signal amplitudes and signal‐to‐noise ratios quantified in spectra and images acquired using the interleaving device were found to be indistinguishable from data acquired in separate, mononuclear acquisitions with the system in its standard configuration.

While the device was demonstrated using ^31^P and ^1^H, the presented method can be used to combine acquisitions of any pair of nuclei the host system is able to excite. The interleaving device has many possible applications, such as ^31^P or ^13^C spectroscopy concurrent with ^1^H spectroscopy or imaging, simultaneous ^23^Na/^1^H imaging, combined ^31^P/^1^H spectroscopic imaging or the use of ^1^H navigators for nonproton MRI. Decoupling at the Larmor frequency of one of the interleaved nuclei can be applied during the readout of the other nucleus (e.g., during an interleaved ^13^C/^1^H acquisition, ^1^H decoupling of the ^13^C readout is possible). However, this effectively reduces the *T*_R_ at the decoupling frequency, which will impact the SNR efficiency for that nucleus. Developing pulse sequences that integrate excitation and readout of signal from two different nuclei is potentially complicated, but the only modification required to use the interleaving device is the addition of a continuous control signal for the duration of the frequency‐shifted acquisition. Most MRI consoles provide such user‐programmable control signals, generally used for triggering and system debugging.

The modification presented here can be applied to any system using superheterodyne receivers where the LO is accessible, where the console allows transmission of RF pulses at different frequencies during a single pulse sequence (as is usually possible on systems equipped with X‐nuclear capabilities for decoupling and nOe), and that provides a digital control signal that can be used to activate the interleaving device during reception.

The method we present here is very similar to the approach recently presented by Jeong and Kaggie 32, 33. Their method introduces an extra mixer, used to shift the frequency of the interleaved ^1^H signal down to the X frequency, which the scanner then records as if it was an X signal. This adds an extra component to the RF chain, which may have some impact on system SNR, but allows all system modifications to be made between the RF coil and the receive plug of the scanner. Our approach is simpler, in that only the receiver LO is modified, with all mixing done by the standard system components in the normal manner. As no additional components are inserted into the receive chain, the impact on SNR is negligible. An advantage of the method presented here is its inherent array coil capability, as all channels of a receiver bank (eight in case of the MRI system used here) are controlled with a single device.

In summary, the device presented in this work enables simultaneous and rapidly interleaved acquisition of signals from two different nuclei, without loss of signal intensity or SNR for either nucleus. The interleaving device is the technical prerequisite for the combining dual‐nuclear imaging and spectroscopy techniques into one sequence, which had been impossible on many clinical platforms, thus opening up exciting possibilities for future research and potential clinical application.
